# Supplementary material for: Are carbon emissions trading and green financial instruments synergistic? -Comprehensive quantitative research based on content analysis
Source: PLoS One. 2024 Mar 7;19(3):e0298601. doi: 10.1371/journal.pone.0298601 (PMC10919601; doi:10.1371/journal.pone.0298601)
Supplement: S1 Data — (ZIP) [file pone.0298601.s001.zip › Data/地级市Z_F工作报告.zip!/╜¡╦╒/─╧═¿╩╨/└┤╫╘╣1⁄2╓┌║┼í╛┬φ┐╦╩2╛▌═°í┐_2009.docx]

各位代表：

　　现在，我代表市人民政府向大会作工作报告，请予审议，并请市政协委员和其他列席人员提出意见。

　　一、2008年工作回顾

　　过去的一年，是很不寻常、很不平凡的一年。面对国际金融危机的严重冲击，面对大事难事较多的复杂局面，全市上下在省委、省政府和市委的坚强领导下，深入贯彻落实科学发展观，按照市委“紧环境下抓机遇，好中求快争一流”的总体要求，同心同德，攻坚克难，在“全面达小康、建设新南通”的征程中迈出坚实步伐。这一年，南通人民百年期盼的苏通长江公路大桥正式通车、洋口深水海港初步通航、吕四港综合海运码头建成，结束了南通“有江无桥”、“有海无深水海港”的历史，南通迈入桥港新时代。这一年，我们积极应对国内外经济环境重大变化，在外部需求明显减弱、宏观经济增速较快下滑的情况下，全力以赴保增长，实现GDP2510.1亿元，按可比价计算增长13.3%；实现财政总收入390.2亿元、比上年增长29.8%，其中地方一般预算收入159.6亿元、增长25%，财政总收入占GDP比重提高1.3个百分点；节能减排约束性目标全面实现；全社会固定资产投资1505.4亿元、增长18.9%；社会消费品零售总额915.1亿元、增长24.2%；城镇居民人均可支配收入18903元，农民人均纯收入7811元，分别增长14.9%和13.1%；城镇登记失业率3.08%；居民消费价格指数涨幅低于全省0.4个百分点；人口自然增长率为-2.5‰。改善民生的12件实事如期完成（见附件一）。这一年，面对特大雨雪冰冻灾害，全市上下众志成城，夺取了抗灾救灾的全面胜利；面对四川汶川特大地震灾情，全市人民倾情相助，彰显了血浓于水的深厚情怀。这一年，我市成功获得全国文明城市、国家历史文化名城、国家园林城市荣誉称号，“五城同创”目标圆满实现。这一年，我们圆满完成了奥运火炬在通传递任务，南通健儿在北京奥运会上勇夺四金一银一铜，在全国地级市中位居第一。

　　一年来，我们主要抓了以下几项工作：

　　（一）突出抢抓桥港机遇，经济发展速度和效益继续高于全省平均水平。放大桥港效应，加快培育新的经济增长点。持续猛攻有效投入，强力推进一批事关全局和长远发展的重大项目，120个市级重点建设项目完成投资417亿元、为年度计划的114.7%；全市工业投入总量破千亿，达1145.7亿元、增长18%，继续保持全省第二。一批龙头型、基地型产业项目快速推进，王子制纸、LNG接收站、熔盛海工基地以及宝钢合金钢技改、通能精机、综艺光伏太阳能等项目开工建设，中远川崎扩建、熔盛造船、晓星超高压变压器、联海生物等项目竣工投产，中远船务海工基地、蓝星化工二期、振华南通产业基地、醋纤五期等项目取得实质性进展。积极有效应对，着力营造区域发展的良好环境。针对年初雨雪冰冻灾害造成的煤电油运紧约束，强化生产要素的调度供应，保障了重点行业、重点企业有序正常运转。针对上半年宏观调控的紧环境，着力化解融资、用地、项目审批等难题，全年新增贷款266.6亿元、增长18%，报批建设用地36046亩，25个重大项目通过国家、省立项审批。针对9月份以来国际金融危机影响不断加深的严峻形势，坚决贯彻中央、省扩大内需的政策措施和市委关于“防下滑、保增长、抓转机、促发展”的决策部署，及时研究出台了扩大内需、促进经济增长的十项措施。应对纺织服装、房地产等行业的下滑趋势以及中小企业的运行困难，有针对性地制定出台26个政策性文件，保持了重点行业和中小企业稳定发展。通过全市共同努力，延续了又好又快发展态势。GDP增幅高出全省0.8个百分点；一般预算收入增幅高出全省2.9个百分点；规模以上工业增加值增幅高出全省2.8个百分点、列沿江八市第一，工业产销率连续四年保持全省第一；进出口增幅高出全省18.4个百分点。各县（市）竞相发展，财政总收入均突破30亿元，通州、海门、启东、如皋四市一般预算收入均突破16亿元、进入全省15强，如东、海安二县一般预算收入增幅均超过30%。省定全面小康的18项25个指标我市预计有20个达标。

　　（二）突出江海联动开发，推动产业优化升级。二三产业增加值占GDP比重达92.1%，提高0.3个百分点。促进沿江沿海优势产业布局。编制完成《南通市沿海开发规划》。进一步推动船舶修造及配套、海洋工程、港口机械、精细化工、新能源、粮油食品等临港临海型产业在沿江沿海集聚。船舶修造及配套产业实现产值629.2亿元、增长54.2%，规模以上造船完工量307万载重吨、增长37.1%；沿海新能源产业发展强劲，已建、在建6个风电项目总装机容量61.2万千瓦。临港临海优势产业的集聚，改变了产业发展对部分传统行业依赖过大的局面，装备制造实现产值突破2000亿元、增长44.4%，精细化工实现产值565.8亿元、增长34.1%，占规模以上工业比重分别提高4.9个、0.5个百分点；促进了企业规模扩张，年销售收入超20亿元的企业达到29家，其中50亿元以上9家；推动了产业集约发展，13个省级以上开发园区以全市3.1%的面积，创造了全市43.2%的GDP、38.2%的财政收入、52.9%的固定资产投资、80.8%的到账外资，投资强度提高到228万元/亩、增长8.5%。扎实抓好自主创新和节能减排。全社会科技投入86.7亿元，其中研发投入36.4亿元。高新技术产业实现产值1438亿元、增长47.2%，占规模以上工业比重提高3.7个百分点。新认定省级高新技术企业88家，新增省级企业工程技术研究中心18家。新增科技软件园区和孵化器面积21万㎡、增长168%。实施重大产学研合作项目230项，培育产学研示范企业556家。获得省科技进步一等奖2项。专利申请量1.41万件、居全省第二。南通家纺市场荣获“世界知识产权组织版权创意金奖”。“人才特区”建设深入推进，新增博士后科研工作站7家、省级引智示范基地2家。加大节能减排力度，COD、SO2排放量分别削减3.86%和11.41%，万元GDP综合能耗下降幅度超额完成省下达目标。连续17年保持耕地占补平衡。成为国家级节水型社会建设试点市。大力推进环境基础设施建设，建成污水处理厂20座，新增日处理能力30.95万吨；90%的发电机组完成脱硫改造，新增脱硫能力2万吨；日处理能力全省第一的如皋垃圾热电联产项目建成投运。大力推进重点行业、重点区域环境整治，提前完成省定关闭150家小化工企业目标。“城市环境综合整治定量考核”连续5年位居全省第一。大力发展服务业。重点发展现代物流、金融、软件、服务外包等生产性服务业，加快发展商贸流通、餐饮、旅游等生活性服务业，服务业增加值增长15%，2002年以来首次超过GDP增幅。全社会物流总量2.68亿吨、增长9.8%，南通港货物吞吐量增至1.32亿吨；市区改建、新建高星级酒店8家；实现旅游总收入150亿元、增长24.2%，获“中国最佳生态旅游城市”称号。

　　（三）突出激发经济活力，进一步扩大对内对外开放。保持开放型经济全省第一方阵地位。加大招商引资力度，先后举办港洽会和深圳、北京、上海、日韩系列招商活动，全市新增工商登记注册外资55.6亿美元，注册外资实际到账29.4亿美元，分别列全省第二、第三；新批项目平均单体规模提高21.5%，新批装备制造和电子信息类项目占比提高2.2个百分点。强力推进外资重点项目，总投资17.5亿美元的35个项目竣工投产，总投资50.1亿美元的102个项目建设进展顺利，现有外资企业累计增资10.3亿美元。转变外贸增长方式，进出口总额166.9亿美元，其中出口117.5亿美元，分别增长30.6%、30.3%，机电、高新技术产品出口占比分别提高4个、3.2个百分点。拓展外经合作领域，完善外派劳务管理机制，新外派劳务1.43万人，新批境外投资项目14个，对外劳务承包营业额等主要指标连续13年保持全省第一。海事、海关、国检、边检等单位整体联动，口岸环境不断改善，如皋港获批为国家一类开放口岸。外事、侨务、对台事务等工作在扩大开放中发挥了积极作用，新缔结国际友城2个。推动跨江联动、接轨上海。在省委、省政府的关心推动下，我市与新加坡、苏州签订合作意向，在苏通大桥北侧联合开发建设中新·苏通生态产业园，这对进一步扩大开放、引领南通新一轮发展具有重要意义。与上海在产业发展、人才引进、产学研合作等方面签订11项战略合作协议，上海外高桥启东产业园开工建设。继续争创江苏民营经济第一大市。个体工商户总数突破30万户，私营企业注册资本突破2500亿元、保持全省第二，民营经济入库税金占全市比重突破60%。做强优势板块，新增销售收入超100亿元特色板块3个，总数达8个；建筑业施工产值突破1500亿元，新增“鲁班奖”4项。做大单体规模，新增营业收入亿元以上民营工业企业134家。做多优势品牌，继续推进“名企名品名人”工程，申报中国名牌产品14个、新增驰名商标8件。

　　注重扩大与央企合作，落户南通的中央国有大企业新开工项目10个，总投资182亿元。注重发挥地方国有资本带动作用，组建南通国有置业集团公司，市属国有资产经营公司资本营运能力日益增强。注重引进更多金融机构落户南通，招商银行、中信银行在通设立分行，上海浦发银行南通支行升格为分行。注重推动企业上市，1家企业成功上市，2家企业通过首发或重组审核，33家企业进入上市轨道。注重事业单位资源整合，市属公益型事业单位改革既定任务基本完成。行业协会、商会作用进一步强化。

　　（四）突出重大基础设施建设，不断提升城市功能。加快构建现代基础设施体系。崇启大桥和海洋铁路开工建设，沪通铁路通过国家立项，兴东机场改造升级规划获批，崇海大桥、宁启铁路宁通段复线电气化改造等前期工作取得重要进展。江海高速、204国道改扩建、334省道改造等工程快速推进，221省道启东和海安段、225省道如东段开工建设。洋口港陆岛通道、太阳岛二期、万吨级重件码头等工程全面完成，吕四港进港航道一期工程竣工，冷家沙海域综合开发前期研究工作启动。西北片引江区域供水工程基本完成，新增受益人口100万人。不断提高城市建设和管理水平。统筹推进中心城市与县（市）城、中心镇建设，修编形成《南通市城市总体规划（2008-2030）》，市区和六县（市）城分别完成城建投入53亿元、46.8亿元。

　　继续拉开市区道路框架，滨江大桥等18项重点工程竣工。加快重点区域建设和改造，新区核心区开工面积超过110万㎡，能达商务区建设启动；老城区污水管网改造加快实施，“名品濠河”建设深入推进；北翼新城建设步伐加快，唐闸、天生港地区保护性改造工程启动。大力发展城市公用事业，实施狼山水厂扩建和居民二次供水设施改造，新增公交车100辆。优化城市环境，市区新增绿地260公顷。全面加强城市管理，市级数字化城管平台基本建成。强力推进文明城市创建，城市形象进一步提升。

　　（五）突出农民增收，着力推进“民富、村美、风气好”的新农村建设。深入推进农民收入倍增工程，农民人均纯收入连续五年保持两位数增长。大力发展现代高效农业。粮食单产创历史新高，高效农业面积和占比继续保持全省领先，“三资”开发农业投入增长30.6%，新建高效农业规模化示范区8个，新增省级以上龙头企业5家。新增无公害农产品48个、绿色食品25个、有机农产品9个，改造中低产田17.75万亩，农业综合开发和农机装备水平进一步提高。大力推动农村综合改革。土地适度规模经营有序推进，新增流转土地6.51万亩。农村三大合作组织新增630家、增长63.5%。组建农村合作银行1家、小额贷款公司2家，农业政策性保险领域和规模不断扩大。大力改善农民生产生活条件。农村新五件实事工程实施情况良好。新增安全饮水人口25万人，新增造林面积34.3万亩、森林覆盖率提高1.4个百分点，农村垃圾集中处理率提高到73%，农村改厕年度任务超额完成。农民集居区规划建设有序推进，新增建筑面积560万㎡，入住农户3.5万户。村级公共服务中心实现全覆盖，新办为农服务社168家。农村文明创建活动深入开展，基层组织建设进一步加强。

　　（六）突出加强公共服务和维护社会稳定，着力保障和改善民生。努力办好社会事业。加大财政对教育的支持力度，城乡免费义务教育全面实施，义务教育债务化解任务全面完成，全市教育财政投入41.27亿元、增长22.7%，增幅高于全市财政经常性收入1.9个百分点。大力推进区域教育现代化建设，积极推动义务教育均衡发展，5个县（市）区达到省教育现代化建设标准。高中阶段教育毛入学率提高5.4个百分点、达92%，高考本一、本二上线率等多项指标列全省第一。南通大学本科教学被教育部评为优秀。中等职业教育基础能力建设进一步加强，南通商贸高等职业学校新校区扩建工程开工。加快完善城乡公共卫生和医疗服务体系，市区社区卫生服务中心实现全覆盖，农村三级卫生服务网络基本形成，传染病发病率稳定下降。加大“无红包医院”创建力度，挂牌成立市区医疗纠纷调处中心，医患关系进一步改善。人口和计划生育工作进一步加强，在稳定低生育水平的同时，严肃查处了一批违法生育行为。进一步推进文化建设。新增国家一级博物馆1家、一级文化馆5家、国家级非物质文化遗产保护项目4个、中国民间文化艺术之乡6个，环濠河文博馆群增加3个新馆。乡镇文化站、村文化室普及达标率分别达94%和79%，新建农家书屋769家。全市有线电视用户新增17.3万户，市区有线电视实现数字化整体转换。“全民健身与奥运同行”主题活动深入开展，成功举办市九运会；8月12日南通健儿在北京奥运会上创造了“一日三金”的历史辉煌，我市设立“南通体育日”。进一步强化城乡就业和社会保障。实施更加积极的就业政策，城镇登记失业率连续10年低于全国、全省水平，90.2%的城镇社区建成充分就业社区；农村劳动力转移新增4.5万人，总数继续保持全省领先。全市城镇基本养老、基本医疗、失业保险覆盖率均稳定在97%以上，新型农村合作医疗参保率达98.3%，市区城镇居民医疗保险基本实现全覆盖。住房保障体系进一步健全，拆迁安置房开工、竣工面积均为历年最多。城乡低保标准继续提高，并为全部城乡低保对象发放物价补贴，农村五保户集中供养率提高4个百分点，农村大病医疗救助全面实施。市区失地农民基本生活保障标准再次提高。老龄事业加快发展，职工权益维护、妇女、儿童、青少年、残疾人、关心下一代、老区扶贫开发、见义勇为、法律援助、红十字和慈善等工作进一步加强。着力维护社会稳定。平安南通、法治南通建设深入推进，矛盾纠纷调处率、刑事案件破案率和社会公众安全感满意率保持全省领先，获省社会治安安全市、社会治安综合治理工作先进市称号，9个县（市）区再次全面进入“社会治安安全县（市）区”行列；“五五”普法通过省中期考核验收。信访渠道进一步畅通，矛盾纠纷排查化解机制和大防控体系、大调解机制不断完善。安全生产事故起数和死亡人数连续7年双下降。社会应急管理体系初步形成。市场价格和食品药品监管扎实有效。国防教育、民兵预备役、优抚安置、人防（民防）、国家安全工作取得新成绩，我市连续13年被省政府评为征兵工作先进单位，实现全国双拥模范城“四连冠”。民族、宗教、地方志、统计、机关事务管理、气象、防震、无线电管理、档案、保密、新闻出版、哲学社会科学等工作得到加强。

　　过去的一年，全市人民全力支援地震灾区抗灾救灾和恢复重建，派出救援人员1000多人次，建成交付过渡安置房2555套，接收并全部治愈伤员99名，接收就读学生303名，社会各界捐款捐物近3亿元，对口支援绵竹新市镇恢复生产、重建家园，第一批总投资9984万元的3个援建项目快速推进。

　　过去的一年，我们积极应对复杂多变的发展环境，不断转变政府职能，努力加强自身建设。进一步深化机关作风建设，继续争创全省最佳办事环境。削减审批事项，推进基建项目网上并联审批和电子政务二期工程，提高服务效能。坚持重大项目办公会和大企业会办会等制度，建立健全“12345”政府服务热线、市长信箱、人民来信来访“三位一体”的服务机制，着力强化对重大项目的推进服务，解决好各类企业发展难题和群众反映强烈的突出问题。完善绩效考评制度，强化目标责任管理，狠抓督办查办，提高落实成效，133项年度重点工作全面完成。加强调查研究，完善科学民主决策机制，28项重点调研课题成果全部转化为解决实际问题的政策意见和工作举措。强化政府法制建设，完善行政复议、应诉制度。深入推进政务公开，抓好行政权力网上公开透明运行。自觉接受市人大及其常委会的法律监督、工作监督和市政协的民主监督，认真执行人大及其常委会决议，支持政协履行职能。高度重视并切实抓好人大代表议案、政协建议案和人大代表建议、政协委员提案办理工作，代表、委员满意基本满意率分别达98.2%、98.4%。坚决落实中央和省关于艰苦奋斗、厉行节约的各项要求，市级机关公用经费支出减少5%。深入开展廉政教育主题活动，强化审计监督，领导干部廉洁自律意识得到增强。

　　各位代表，回顾过去的一年，所取得的成绩是省委、省政府和市委正确领导的结果，是全市人民和衷共济、奋力拼搏的结果。在此，我谨代表市人民政府，向广大干部群众，向人大代表、政协委员和离退休老同志，向各民主党派、工商联、各人民团体和各界人士，向人民解放军、武警驻通部队官兵、公安干警和部、省驻通单位，向所有关心和支持南通发展的海内外朋友，致以崇高的敬意和衷心的感谢！

　　各位代表，回顾过去的一年，我们也深深地感到，政府工作中还存在不足，社会公共管理还存在薄弱环节，一些政府工作人员服务意识和能力还有待增强，在政府系统还存在不正之风甚至腐败现象。同时，我们还清醒地看到，自主创新能力和产业竞争力还不强，转变经济发展方式的任务还很重；经济发展面临的深层次矛盾还比较多，制约我市经济发展的体制性、结构性矛盾依然存在，经济增长的要素、资源瓶颈制约仍较突出；经济下行压力加大，工业增速回落，部分企业生产经营困难、经济效益下滑，财政减收增支因素明显增多；就业形势趋紧，城乡居民增收难度加大，部分居民生活比较困难，保障民生、维护社会稳定的任务更加繁重。对此，我们将始终保持清醒头脑，时刻忧患在心，充分准备，积极作为，尽最大的努力，争取最好的结果。

　　二、2009年目标任务

　　今年是新中国成立60周年，也是我市实现全面小康目标的决胜之年。做好今年政府工作，意义十分重大。尽管国际金融危机对我国经济的影响还在加深，今年将是本世纪以来经济发展最为困难的一年，但应看到，国家实施扩大内需的一系列政策措施为我们创造了更为宽松的发展环境；长三角一体化进程进一步加速，江苏沿海开发上升为国家战略，特别是大桥大港的建成，为我们提供了更多的发展机遇；南通多年形成的产业基础以及政通人和的良好发展氛围，都极大增强了我们应对挑战风险的能力，南通已站在一个新的历史发展起点上，“危中更有机、难中可攀高”，我们对“扎实保增长、全面达小康”充满信心。

　　今年政府工作总体要求是：以邓小平理论和“三个代表”重要思想为指导，以科学发展观统揽全局，全面贯彻党的十七大、十七届三中全会精神，按照省委、省政府和市委一系列决策部署，牢牢把握“扎实保增长、全面达小康”工作主线，把保持经济平稳较快发展作为首要任务，把在江北率先以市为单位实现全面小康作为决胜目标，力争以县为单位实现全面小康建设“满堂红”，抢抓桥港时代新机遇，把握国家扩大内需新契机，坚定信心，难中攀高，加快经济结构调整和发展方式转变，提高国际竞争力；加大城乡区域统筹力度，提升协调发展水平；深化改革开放，增强发展活力和动力；切实改善民生，维护社会和谐稳定，全力推进经济社会又好又快发展。

　　综合考虑各种因素，今年全市经济社会发展的主要预期目标是：GDP增长11%、力争12%；完成或超额完成省下达节能减排工作指标；地方一般预算收入增长12%以上；全社会固定资产投资增长17%以上；外贸进出口总额增长10%以上；社会消费品零售总额增长16%；城镇居民人均可支配收入、农民人均纯收入分别增长10%和9%；居民消费价格指数涨幅控制在4%左右；城镇登记失业率控制在4%以内；人口自然增长率控制在零左右。

　　围绕上述要求和目标，我们将重点抓好六个方面工作：

　　（一）把扎实保增长作为首要任务。千方百计扩大内需，培育新的增长点，确保主要经济指标增幅继续高于全省平均水平。

　　加大有效投入保增长。重点抓好90个总投资1279亿元的续建项目、90个总投资856亿元的新开工项目、90个总投资2525亿元的前期项目，力争市级重点建设项目完成投资529亿元。加快重大基础设施项目建设，力争沪通铁路、宁启铁路宁通段复线电气化改造早日开工；抓好崇启大桥、海洋铁路、江海高速和洋口港、吕四港港区设施建设，兴东机场升级改造，以及204、328国道和221、225、334、226省道改扩建；做好崇海大桥、宁启铁路通启段、如皋港铁路、海启高速、临海高等级公路等项目前期工作；推进焦港船闸建设和电力过江通道规划建设。加快重大产业项目建设，重点推进中远船务海工基地、中集大型储罐等项目早日投产，王子制纸、LNG接收站、吕四大唐电厂、熔盛海工基地、振华港机重型钢结构等项目加快建设，华能和天电“上大压小”、海门国电、蓝星化工二期、醋纤五期等项目尽快获批开工；扎实抓好总投资271亿元的70个市级服务业项目，尽早启动沙钢冶金物流园、沿海煤炭集配基地等生产性服务业项目，快速推进南通大饭店扩建、中南国际广场、润华国际中心等13个高星级酒店项目。加快重大民生工程项目建设，重点推进市民服务中心、社会福利中心、图书馆新馆、报业新闻传媒中心、第六人民医院、中医院综合楼等项目。加强对政府投资的监管，引导和扩大社会投资，提高投资质量和效益。

　　扶持企业保增长。积极落实并指导企业用好财税、信贷、出口等各类扶持政策，有针对性地帮助企业救急解难。减轻企业运行负担，实施增值税转型改革让利12亿元，落实60项减免税费措施，全面清理涉企收费，帮助企业增强信心、渡过难关。拓宽企业融资渠道，进一步完善银政企合作机制，引导金融机构多授信、多放贷，力争各类金融机构贷款增幅高于全省2个百分点以上；帮助企业加大短期融资券、中期票据的融资力度，积极推动发行企业债券。扶持中小企业和优势企业发展，设立总规模3亿元的创业投资引导基金，充实扩大中小企业互助基金规模，加大对中小企业的扶持力度；市本级财政安排3亿元企业发展专项资金，全市重点培育新增销售收入5000万元以上企业200家，扶持1500家高增长、有品牌、有市场、自主创新能力强的企业加快发展，力争新增规模以上企业200家、总数突破6000家，其中新增销售收入超亿元工业企业50家、总数超过1100家。

　　扩大消费保增长。增加收入促进消费。落实有利于就业、创业的财税、担保等扶持政策，加大社会保障力度，提高中等收入者比重和困难群众生活补助，增加城乡居民工资性、财产性和经营性收入。培育市场拉动消费。加大政策落实力度，统筹推进保障性住房、普通住房、商办用房建设，积极搞活二手房市场和住房租赁市场，全力保持房地产业平稳健康发展。稳定汽车等大宗消费，大力发展社区商业、物业、家政等服务性消费，积极开发旅游、文化、体育健身和创意产业等热点消费，促进消费结构升级。优化环境推动消费。鼓励和引导诚信消费，规范和发展信贷消费。建立健全流通网络和服务体系，大力推进“万村千乡”市场工程和“家电下乡”工作，积极开拓农村消费市场。

　　（二）紧扣全面达小康目标，统筹城乡、区域协调发展。全面达小康是市第十次党代会的重大决策和本届政府的郑重承诺。我们要建设的全面小康是不含水分、群众得实惠、老百姓认可的高水平全面小康。全面达小康，重点在县（市），难点在农村。大力发展县域经济。支持已经达到全面小康标准的县（市）对照更高要求，巩固成果，提升水平；帮助今年计划达标的县（市）对照薄弱环节，加大攻坚力度，力争如期达标。促进各县（市）依托自身特色资源，放大比较优势，做大做强特色经济。强化对城镇建设的分类指导，促进县（市）城向现代化中等城市发展，加强重点中心镇培育，加快建设一批临港新镇、工贸强镇、旅游名镇，增强城镇综合承载能力和农村发展带动能力。深入推进“民富、村美、风气好”的新农村建设。落实惠农强农政策，增加公共财政对“三农”的投入，促进农业不断增效、农民持续增收、农村和谐发展。深入实施高效农业规模化“133行动计划”和设施农业“863”工程，新增净收益2000元以上高效农田25万亩，其中设施农业7万亩。大力发展品牌农业，建成省级农业标准化示范区4个。健全农村服务体系，强化农业资源综合开发，提升农业技术和农机装备水平，主要农作物生产综合机械化水平达80%以上。坚持农村基本经营制度，鼓励和促进土地承包经营权依法流转，推进“万顷良田建设工程”试点和农业适度规模经营。大力发展农村三大合作组织，提高农民组织化程度。落实国家和省要求，增加对种粮农民直接补贴、良种补贴、农机具购置补贴、农资综合补贴。大力发展村级集体经济，严格控制村级负担，积极化解村级债务。扩大村民自治范围，加强农村新社区建设，提升农村文明程度。抓好扶贫和老区开发。着力统筹城乡发展。加快城乡规划、产业布局、基础设施、公共服务和劳动就业“五个一体化”，促进城乡共同进步。办好新一轮农村六件实事，加强农村道路、供电、供水、通信、环境等基础设施建设，全面发展农村教育、文化、卫生等社会事业。深入推进生态市创建，加强农村面源污染治理和环境综合整治，完善生活垃圾统一收集、集中处理机制，改造农村厕所15.6万座；推进“四沿”绿化和村庄绿化，加强农田林网建设，力争森林覆盖率达20%；开工建设15个污水处理项目、新增日处理能力20万吨，生活污水集中处理率提高5个百分点；抓好饮用水源地环境保护，确保功能区水质达标；推进海洋环境综合整治；加强基层生态创建，力争30个镇基本达到全国环境优美乡镇考核标准。

　　（三）提升江海联动开发的层次和水平，促进产业升级转型。加大沿江沿海优势产业布局力度。认真实施《南通市沿海开发规划》，推动生产力布局向沿海拓展，重点发展能源、石化、冶金、现代物流等产业。健全江海优势产业发展平台的共建共享机制，促进企业、项目向省级以上开发区和重点产业园区集中。加强江海资源的深度整合和综合利用，科学利用岸线、滩涂资源，重点加快新通海沙围填整治和长江北支、天生港水道整治开发，抓好横港沙、如皋沙群开发利用和冷家沙海域综合开发前期工作，推进洋口港北水道20万吨级航道和吕四港10万吨级航道研究设计。提升先进制造业发展水平。调高层次，推进主导产业高端化。围绕装备制造、电子信息、精细化工等主导产业，促进产业链向高端攀升。认真落实国家装备制造业振兴计划，重点发展超大型船舶、特种工程船舶修造及配套和海洋工程、港口机械、电力设备、金属制品等，力争产值占规模以上工业比重提高2个百分点。调大总量，推进新兴产业规模化。大力发展新材料、新能源、新医药以及环保等新兴产业，实现产值占规模以上工业比重提高到5%。调优质态，推进传统产业品牌化。积极运用先进适用技术改造传统产业，打造纺织服装、粮油食品、冶金建材等行业优势品牌，重点发展家用纺织、现代服装服饰、高档面料，提高市场竞争力。积极推进自主创新和节能减排。力争高新技术产业产值占规模以上工业比重达29%。加大研发投入，培育科技创新主体，新增市级以上企业工程技术研究中心250家。实施重大产学研合作项目200项，新增科技孵化器面积100万㎡。加强具有产业特色的公共技术服务平台建设。办好“人才特区”，更多引进高层次、高技能人才。深入推进百家重点用能企业节能工作和节能减排全民行动。继续实施节能和循环经济“双百”工程、“310”资源节约和清洁生产示范工程。抓好36个已建污水处理厂的管网拓展工程，提高实际处理负荷率。市区实现姚港化工区三类企业全面停产，加快钢丝绳热处理中心建设。坚持最严格的耕地保护和节约用地制度，提高土地资源利用效率。更大力度推动服务业发展。力争服务业增加值增幅高于GDP增幅1个百分点。重点发展现代物流、金融保险、服务外包、商务会展等生产性服务业。加快重点物流园区规划建设，扶持现代物流大企业发展，提高物流增加值，力争全社会物流总量达2.7亿吨。大力发展滨海、乡村、地域文化等特色旅游，加快狼山、濠河创建5A级景区步伐，推进苏通大桥观景台、陈氏花园等一批旅游项目建设，着力打造长三角新兴旅游休闲胜地。积极推进21个服务业集聚区和区域性大型农产品批发交易中心建设。优化服务业发展环境，加大财税、规划、土地、用房、价格等政策支持，引进更多优质品牌，更新服务业态，加速服务业提档升级。

　　（四）进一步扩大开放、深化改革。快速启动、扎实推进中新·苏通生态产业园开发建设。加快建立高层协调推进机制，提升合作开发模式，编制开发园区规划，力争5月份奠基开工，并快速启动核心区建设。以加强与上海和苏南开发园区跨江合作为重要抓手，全力推动开发园区争先晋位、主要指标在全国全省排名前移，力争13个省级以上开发园区GDP、财政收入和实际利用外资占全市比重分别提高2个、4个、2个百分点。着力提高招商选资水平。更加注重引进产业关联度高、上下游配套能力强的优势项目，重点推进能够发挥南通比较优势的先进制造业、现代农业、生产性服务业招商。更加注重引进龙头型、基地型、带动力强的重特大项目，力争引进总投资1亿美元项目5个、全球500强投资项目10个。更加注重瞄准重点区域、重点企业招商，紧盯日本、韩国、东南亚、台湾等区域，紧盯世界级、国字头大型企业，紧盯现有外资企业，精准化出击，确保注册外资实际到账20亿美元。更加注重专业队伍建设，加强人才配备和技能培训，提高招商的有效性和成功率。努力保持进出口稳定增长。充分利用国家提高出口退税率和推进增值税转型改革的契机，增强船舶、化工、家纺等行业出口产品竞争力，提高机电和高新技术产品出口比重，重点培育出口超1000万美元大户200家，巩固传统市场，开拓新兴市场。抓住国际大宗商品价格处于相对低位的有利时机，扩大国外先进技术、关键装备和紧缺资源进口。推进劳务分包向工程总承包转变，保持外经营业额稳定增长。加快实施“走出去”战略，力争新批境外投资企业15家。不断提升民营经济发展层次。继续争创江苏民营经济第一大市，推动全民创业，保持私营企业总数及注册资本全省第二。着力培育板块经济，力争新增超百亿元板块2个，每个县（市）培育特色产业板块3-5个。以南通家纺城、海门叠石桥等专业市场建设为重点，打造具有国际竞争力的家纺板块。利用国家扩大内需和建筑市场重新洗牌的契机，打造全国一流建筑强市，实现施工产值1700亿元以上。着力放大“三名”效应，争创中国名牌和驰名商标10件以上，力争营业收入超50亿元民营企业达10家。着力推动企业上市和优势企业购并重组。推进民营经济国际化，鼓励民营企业“走出去”。深入推进重点领域和关键环节改革。贯彻实施《企业国有资产法》，推动国有企业产权制度和管理体制创新，完善风险防范和内控机制。加快投融资体制改革，引导和鼓励民间资本扩大投资领域。深化农村合作金融机构改革，推动农村信用社组建商业银行。推进小额贷款公司县（市）全覆盖，扩大农村抵押担保贷款范围，完善农业政策性保险制度。全力支持金融机构做大做强，鼓励和吸引更多的市外金融机构落户南通。进一步探索“管办分离”路子，巩固公益型事业单位改革成果。

　　（五）增强中心城市集聚和辐射功能。着力做大做强市区，放大与上海的同城效应，充分发挥中心城市对各县（市）的辐射带动作用。进一步优化市区空间结构。按照“两轴三区四带”城市空间结构要求，深化和完善城市总体规划、综合交通规划、历史文化名城保护规划，加强城乡结合部规划控制和引导。加快新城区建设，重点抓好中央商务区、开发区能达商务区建设，引导优质资源向新区集聚，促进新区东拓、南接、西连和北优。提升老城区环境品质，启动南大街地下空间利用工程，推进3个片区危旧房改造和4个老小区综合整治，开工改造10个“城中村”。以北城大桥开工建设为契机，加大长华路两侧和火车站站前区开发建设力度，加快形成北翼新城核心区雏形。进一步完善中心城市功能。组织实施100个城建项目，力争投入突破100亿元。着力增强城市交通功能，重点围绕“便捷、快速、顺畅”目标，以绕城高速为依托，开工建设通沪大道高架、东快速路等6项工程，加速构建“一轴一环八射”快速路系统，实现30分钟出城；拉开道路框架与完善路网结构并重，加快骨干道路和支路建设，大力发展城市公交，优化道路交通组织管理，改造重点交通节点，缓解老城区部分地段拥堵问题。着手开展市区轨道交通前期研究。完善市区污水处理、供水、照明、停车等公共设施。进一步放大“五城同创”效应。以推进数字化城管为手段，以加强交通秩序管理、车辆停放管理、小区物业管理为重点，健全“五城同创”长效管理机制，全面提高城市综合管理水平。打造“名品濠河”，加大濠河周边地区环境整治和功能配套力度；开辟濠河引水第二通道，疏浚整治市区河道12条段。市区新增绿化面积300公顷。抓紧实施寺街·名人文化区、南通·1895等历史文化保护工程，彰显近代文化特色，体现地方传统风貌，提升城市文化品位，推动“中国近代第一城”焕发新气象。

　　（六）更好保障和改善民生，加强社会建设和管理。着力促进就业。落实社会保险补贴和税收减免政策，鼓励生产企业和服务业更多吸纳就业，力争新增城镇就业7万人。推进充分就业社区创建，加强对下岗失业人员、返乡农民工、高校毕业生的培训，鼓励自主创业、自谋职业，以创业带动就业。加大财政购买公益性就业岗位和就业援助力度，完善零就业家庭动态清零长效机制。加强村级就业服务体系建设，促进农村劳动力转移就业。健全完善覆盖城乡居民的社会保障体系。进一步扩大城镇职工基本养老保险、基本医疗保险覆盖面。推动农民工参加工伤保险和医疗保险，力争工伤保险实现市级统筹。完善失业保险制度，及时做好关闭破产企业新失业人员、退休人员的生活保障。进一步完善被征地农民基本生活保障制度。完善住房保障体系，加快市区拆迁安置房、廉租住房、经济适用房、外来务工人员宿舍等保障性住房建设，确保投入不少于40亿元，对申请实物配租的低保住房困难家庭“应保尽保”。着力构建城乡新型救助体系，建立市区城镇老年居民养老补贴制度，加强临时和应急救助，提高农村五保户供养水平。进一步提高城乡低保补助标准，提高企业退休人员养老金以及优抚对象等人员抚恤和补助标准。支持慈善会和红十字会工作，鼓励慈善捐助和人道救助事业。继续实施脱贫攻坚工程，力争农村贫困人口减少10万人。更加重视发展老龄和残疾人事业，深入开展法律援助，保障妇女儿童和弱势群体合法权益。全力维护社会平安稳定。以“长安”新理念，严格领导责任、部门责任和单位责任，全面落实维护社会平安稳定的各项措施。大力加强社会治安综合治理，强化城乡社区建设、服务和管理，进一步巩固社会矛盾纠纷大调解机制和社会治安大防控格局，严厉打击各种违法犯罪活动，强化防范和处理邪教工作，提升平安南通、法治南通建设水平。高度重视做好新形势下的信访工作，强化领导干部下访和包企包案工作，定期排查、及时发现和处理苗头性问题，有效防范企业停产、征地拆迁、工程欠款、涉法涉诉等问题引发的群体性事件。加强外来人口服务管理。健全完善应急管理机制，加强社会应急资源整合，增强对重大突发事件的处置能力。严格安全生产责任制，强化道路交通、海洋渔业、化工、消防、建筑等领域专项整治；突出企业业主安全生产责任制，严厉打击各种违法违规生产经营行为，坚决防止重特大安全事故。进一步整顿和规范市场秩序，切实做好价格监管和市场调控。健全食品安全长效管理机制，提高食品动态安全监测水平。加快发展社会事业。积极推动教育高位持续发展。制定教育中长期改革发展规划，进一步落实促进教育发展的有效措施。完善教育投入保障机制，推动“规范教育收费示范县（市）区”创建。加快区域教育现代化进程，力争全市基本达到区域教育现代化标准。深入实施素质教育，推进义务教育均衡发展，在义务教育学校实施绩效工资。所有普通高中达到三星级标准。加快特殊教育合格学校建设。力争60%的幼儿园建成省优质幼儿园。优化职业教育专业设置，加快商贸高职新校区建设和工贸技校扩建，提升职业教育发展水平。积极支持南通大学内涵发展。坚持基本医疗的公益性质，有序推进基本公共卫生服务均等化，推动公立医院补偿机制改革，重点办好县级医院、农村社区卫生服务中心，健全完善农村三级医疗卫生服务网络，强化重大疾病预防控制，市区全面实行社区卫生服务机构基本药物销售零差价。积极推进中医药事业健康发展。加快发展妇幼卫生事业。加大“无红包医院”创建力度，构建和谐医患关系。推进乡镇文化站、村文化室和“农家书屋”全覆盖，市区新建文艺之家。围绕庆祝新中国成立60周年，创作一批精品力作。加强历史文化遗产和非物质文化遗产保护。加强文化对外交流合作，承办尼泊尔第四届“中国文化节”活动。扶持文化产业发展。启动县（市）有线电视数字化整体转换工作，力争完成率超过30%。推进全民健身中心建设和市儿童业余体校训练馆改建工程，认真组织全民健身月和南通体育日系列活动。完善计划生育利益导向机制，继续稳定低生育水平。做好第二轮修志工作。继续加强哲学社会科学、关心下一代、统计、档案、无线电管理、保密等工作。进一步提升社会文明程度。以成功创建全国文明城市为新起点，继续弘扬精神文明“南通现象”和新时期南通精神，大力开展群众性精神文明创建活动，深入实施公民道德建设工程和公民素质提升计划，加强社会公德、职业道德、家庭美德和未成年人思想道德建设。进一步加强基层民主建设，保障人民依法行使民主权利。继续加强民族、宗教、侨务、对台、人防（民防）、国防教育、征兵、民兵预备役、“双拥”、优抚安置、国家安全、机关事务管理、气象、防震减灾等工作。

　　各位代表，今年我们将继续集中财力物力，为民办好一批实事。各级财政安排28.49亿元，带动社会投资42.17亿元，兴办医、食、住、行、就业、养老、区域供水等方面的12件26项实事（见附件二），努力让发展成果实实在在惠及更多老百姓。

　　各位代表，难得的机遇和严峻的挑战对政府工作提出了更高要求。我们将以开展学习实践科学发展观活动为动力，进一步转变政府职能，更加负责地担当起“扎实保增长、全面达小康”的重任。大力提升工作创造力。不断解放思想，强化服务经济、服务企业的能力，把中央精神和地方实际紧密结合好，有针对性地完善扶持产业发展、推进项目建设的政策举措，支持开发园区实行精简高效的管理模式；创新重大项目领导办公会、重点企业会办会等机制，以解决实际问题作为检验部门服务成效的标准，更加扎实有效地帮助化解好立项报批、项目用地、企业融资、征地拆迁等难题；优化行政审批流程，推进网上并联审批，强化限时办结，促进审批提速。强化服务群众的能力，提升“12345”政府服务热线、市长信箱、人民群众来信来访的服务质量，加强各类服务平台资源整合、整体联动。继续推进政企分开、政资分开、政事分开、政府与市场中介组织分开，充分发挥行业协会、商会作用，努力为市场主体创造良好发展环境、提供优质公共服务。切实增强行政执行力。强化督办查办，加大重点工作、重大项目和重要民生问题的跟踪督查力度，确保政令畅通。强化行政问责，加大责任追究力度。强化绩效考核，探索建立科学的政府绩效评估体系和经济社会发展综合评价体系，加大绩效审计和效益审计力度。强化调查研究，政府重要政策、重要决策、重大事项，特别是涉及民生的重大问题，没有深入调研不决策，没有方案比选不决策，没有科学论证不决策。不断提高政府公信力。坚持依法行政、阳光行政、廉洁行政，树立“为民、务实、清廉”的良好形象。进一步落实国家和省关于加强市县政府依法行政的法规要求，严格按法定权限和程序履行职责，推进政府各项行政管理规范化、法治化。深化政务公开，完善信息公开和新闻发布制度，加快电子政务二期工程建设，确保行政权力网上公开透明运行年内推进到位，切实保障公众的知情权、参与权、表达权和监督权。深入贯彻《监督法》，自觉接受人大及其常委会的监督，主动接受政协的民主监督，广泛听取各民主党派、工商联、无党派人士意见，充分发挥工会、共青团和妇联等人民团体在构建和谐社会中的积极作用，主动接受舆论监督和社会监督。高度重视办好人大代表议案、政协建议案和人大代表建议、政协委员提案。建立健全惩治和预防腐败体系，坚持从源头上惩治和预防腐败；加强对权力运行特别是领导干部、人财物管理使用、关键岗位的监督和制约，推行政府投资项目代建制；深入开展专项治理，坚决纠正损害群众利益的不正之风；严肃查处各类违法违纪案件；坚持艰苦奋斗、厉行节约，建设节约型机关；加强对公务员和各级领导干部的教育、监督、管理，建设一支廉洁、勤政、务实、高效的公务员队伍。

　　各位代表！做好今年工作，任务艰巨而繁重，责任重大而光荣。让我们紧密团结在以胡锦涛同志为总书记的党中央周围，高举中国特色社会主义伟大旗帜，在省委、省政府和市委的正确领导下，解放思想，坚定信心，团结拼搏，迎难而上，全力开创我市又好又快发展新局面，以“扎实保增长、全面达小康”的优异成绩向新中国成立60周年献礼！
